# Epidemiology of Multidrug-Resistant Microorganisms among Nursing Home Residents in Belgium

**DOI:** 10.1371/journal.pone.0064908

**Published:** 2013-05-30

**Authors:** Béatrice Jans, Didier Schoevaerdts, Te-Din Huang, Catherine Berhin, Katrien Latour, Pierre Bogaerts, Claire Nonhoff, Olivier Denis, Boudewijn Catry, Youri Glupczynski

**Affiliations:** 1 Epidemiology Unit, Scientific Institute of Public Health, Brussels, Belgium; 2 Department of Geriatric Medicine, CHU UCL Mont-Godinne-Dinant, Yvoir, Belgium and Institute of Health and Society, Catholic University of Louvain, Public Health School, Brussels, Belgium; 3 National Reference laboratory for monitoring of Antimicrobial Resistance in Gram-negative bacteria, Department of Clinical Microbiology, CHU UCL Mont-Godinne-Dinant, Catholic University of Louvain, Yvoir, Belgium; 4 National Reference Laboratory of MRSA and Staphylococci, Department of Clinical Microbiology, Hôpital Erasme, Université Libre de Bruxelles, Brussels, Belgium; Amphia Ziekenhuis, The Netherlands

## Abstract

**Objectives:**

A national survey was conducted to determine the prevalence and risk factors of methicillin-resistant *Staphylococcus aureus* (MRSA), extended-spectrum β-lactamases-producing Enterobacteriaceae (ESBLE) and vancomycin-resistant enterococci (VRE) among nursing home residents in Belgium.

**Methods:**

A random stratified, national prevalence survey was conducted in nursing home residents who were screened for carriage of ESBLE, MRSA and VRE by multisite enriched culture. Characteristics of nursing homes and residents were collected by a questionnaire survey and were analysed by multilevel logistic regression analysis.

**Results:**

Of 2791 screened residents in 60 participating nursing home, the weighted prevalence of ESBLE and MRSA carriage were 6.2% (range: 0 to 20%) and 12.2% (range: 0 to 36%), respectively. No cases of VRE were found. No relationship was found between ESBLE and MRSA prevalence rates within nursing homes and the rate of co-colonization was very low (0.8%). Geographical variations in prevalence of MRSA and ESBLE and in distribution of ESBL types in nursing home residents paralleled that of acute hospitals. Risk factors of ESBLE carriage included previously known ESBLE carriage, male gender, a low level of mobility and previous antibiotic exposure. Risk factors for MRSA colonization were: previously known MRSA carriage, skin lesions, a low functional status and antacid use.

**Conclusions:**

A low prevalence of ESBLE carriage was found in nursing home residents in Belgium. The prevalence of MRSA carriage decreased substantially in comparison to a similar survey conducted in 2005. A low functional status appeared as a common factor for ESBLE and MRSA carriage. Previous exposure to antibiotics was a strong predictor of ESBLE colonization while increased clustering of MRSA carriage suggested the importance of cross-transmission within nursing homes for this organism. These results emphasize the need for global coordination of the surveillance of MDRO within and between nursing homes and hospitals.

## Introduction

Infections due to multidrug-resistant bacteria are a major health concern worldwide [1]. Asymptomatic colonization by multidrug-resistant microorganisms (MDRO) has been recognized as the first step before subsequent infection [2], [3], [4]. Moreover, infections due to MDRO have been associated with a delay in initiating effective therapy, a higher mortality, and an increase of the length of hospital stay with subsequent increases of medical costs [5], [6]. Nursing homes (NHs) may represent a large reservoir of MDRO since these institutions do admit old frail residents who frequently require a higher degree of medical care and often need to be hospitalized. In 2005, a Belgian cross-sectional survey showed that on average 19% of the screened NH residents were methicillin-resistant *Staphylococcus aureus* (MRSA) carriers [7]. Studies in various countries have also reported, among NH residents, high rates of asymptomatic colonization by other MDROs like extended-spectrum β-lactamase producing Enterobacteriaceae (ESBLE) and vancomycin-resistant enterococci (VRE) [8], [9].

The aim of the present study was to determine the prevalence and potential risk factors of colonization with three selected MDROs, namely MRSA, ESBLE and VRE in a large cohort of NH residents.

## Materials and Methods

### Ethics Statement

The study protocol was approved by the Ethical Committee of CHU UCL Mont-Godinne (National number: B03920109042). Written informed consent was obtained from each resident enrolled in the study, or from their legal representatives in case of cognitive disorders. All data were reported anonymously with regard to patient and NH identification. Microbiological results of residents were confidentially notified to their family doctor.

### Study design

A cross-sectional prevalence survey was conducted from June to October 2011. Sixty NHs (5.3%), with a total of 5608 beds (median 94 beds; range 31–187 beds), were selected from the national insurance database [7]. The selected NHs were equally representative by region (Flanders, Walloon region and Brussels), by size and by the proportion of high-skilled beds in the NHs.

Residents were accommodated in rooms with one to four beds. On site, the study coordinator randomly selected up to 50 residents (and 10 reserve) from the residents' registry according to a previously described methodology [7]. In case of accommodation in rooms of more than one bed, all roommates of selected residents were screened for carriage of MDRO.

Taking into account the cluster effect and an alpha level of 0.05, to achieve an absolute precision of estimate of ±2% with a confidence level of 95% and an expected prevalence of 20% for MRSA, 8% for ESBLE producers and 5% for VRE, a sample size of 3000 residents was calculated.

### Data collection

In each facility, one reference nurse and one coordinating physician coordinated the survey. For each participants, a structured questionnaire had to be completed in order to collect the following data: demographic and administrative data (including the number of roommates), length of stay in the facility at time of sampling (months), autonomy in basic activities of daily living according to the modified Katz scale including five levels from less dependent to highly dependent (category O, A, B, C, CD), mobility status (ambulant or wheelchair-bound and bedridden), urinary and/or faecal incontinence, presence of wounds or decubitus ulcer, indwelling urinary catheter, percutaneous gastrostomy, nasogastric tube feeding, antacid or corticoid use, current or previous antibiotic exposure within prior three months, hospital stay during the last 12 months, recent surgery (last 3 months) and previously or currently known MRSA or ESBLE carriage/infection (last 12 months). Underlying diseases were assessed using the Charlson's Comorbidity Index and categorized in three groups (no or mild, moderate, severe) [10]. The NHs were categorized in three different types according to the proportion of high-skilled beds: low care (<45%), medium care (between 45–65%) and high care (>65%).

### Microbiological analysis

In each NH, local nurses performed a same-day sampling of two series of swabs including a first set pooling anterior nares, throat, perineum and chronic wound for the detection of MRSA detection (Kit MRSA trypticase soy broth supplemented with 2.5% NaCl, Copan innovation, Brescia, Italy) and a second set consisting in a rectal swab (ESwab, Copan innovation, Brescia, Italy) for the detection of ESBLE and of VRE. All specimens were sent to a central laboratory. After 24 h incubation, broths were subcultured onto chromogenic *Brilliance™* MRSA-2 Agar (Oxoid, Hampshire, UK). Rectal swabs were incubated overnight in brain-heart infusion broth and were thereafter subcultured onto three selective media, including MacConkey (with ceftazidime disk (30 µg)), selective chromogenic *Brilliance™* ESBL Agar (Oxoid, Hampshire, UK) and *Brilliance™* VRE Agar (Oxoid, Hampshire, UK). Rectal swab specimens which did not yield any bacterial growth on MacConkey agar following broth enrichment culture were considered of insufficient quality and were excluded from further analysis.

Bacterial identification of suspected colonies was carried out by MALDI-TOF mass spectrometry (Bruker, Leipzig, Germany). Oxacillin susceptibility for *S. aureus* was tested by cefoxitin disc (30-µg) according to the recommendations of the Clinical Laboratory Standard Institute (CLSI) [11]. MRSA were confirmed by multiplex PCR for *nuc*, *mecA* and 16S rDNA genes [12]. For gram-negative bacteria, confirmation of ESBLE was carried out by double disc combination synergy test with cefotaxime and ceftazidime with and without clavulanic acid according to CLSI guidelines [11]. *In vitro* susceptibility of ESBLE isolates was determined by Vitek-2 automate using AST-N156 cards (bioMérieux, Marcy-L'Etoile, France) with EUCAST interpretative breakpoints. Characterization of β-lactamase genes was performed by two triplex end-point PCR assays targeting *bla*_TEM_, *bla*_SHV_, *bla*_OXA-1_ and *bla*_CTX-M_ of group 1, 2 or 9 and/or by a commercial ESBL/plasmidic AmpC DNA low-density microarray (Check-MDR CT101; Check-Points, Wageningen, The Netherlands). Vancomycin susceptibility for *Enterococcus* spp. was determined by Vitek-2 (AST-P586 cards) and multiplex PCR for detection of *van* genes was performed as, previously described [13].

### Statistical analysis

For categorical variables, the degree of association was measured by the Chi-square or by Fisher's exact test when appropriate. Numerical data were compared by Student's *t* test for normally distributed data and Wilcoxon Rank-Sum test in other cases. All tests were two-tailed and were performed by Stata 12.1 SE (StataCorp LP, Texas, USA). Prevalence was calculated using the cluster survey analysis module. Weighted prevalence referred to the prevalence adjusted for the participation rate in each NH. 95% confidence intervals (95%CI) were calculated using the Poisson distribution. Odds ratio (OR), provided in univariate analysis, were calculated by logistic regression analysis. In multivariate analysis, the dependant variable was the presence or absence of one of each isolated microorganisms of interest (MRSA, ESBLE or both) from at least one screened site. All predictors in univariate analysis with a *P*-value under 0.05 are reported in Table 1. All potential predictors were further included in the multivariate models built using stepwise logistic regression with backward selection (*P*-value≤0.10) of variables by the likelihood ratio test. A random-effect logistic regression analysis was used to adjust for multiple risk factors and for the clustering of bacteria carriage among NHs. The model used two levels of hierarchy, placing the individual patient-related risk factors at the first level, named the “patient level,” and the NHs in the second level (named “NH level”), representing the “NH effect.” Using multilevel regression model, the intercept of each regression line was allowed to vary at random between NHs (random intercept). The variance attributable to the NH level was estimated with the Intraclass Correlation Coefficient (ICC).

**Table 1:** Risk factors of colonization by ESBLE or MRSA among a random sample of residents screened within 60 nursing homes in Belgium: results from univariate analysis.

Predictors	ESBLE Carriers (n = 2610) Unadjusted OR (95%CI); *P*-value	MRSA carriers (n = 2789) Unadjusted OR (95%CI); *P*-value
Male gender	1.4 (1.0–3.0); 0.032	1.4 (1.1–1.8); 0.015
Length of stay in NH >24months	0.8 (0.6–1.1): 0.278	1.3 (1.0–1.6); 0.048
Rooms with ≥2 beds	1.0 (0.7–1.4); 0.984	1.4 (1.1–1.8); 0.006
Modified Katz score C or CD	1.7 (1.2–2.3); 0.001	1.6 (1.3–2.0); <0.001
Mobility level: wheelchair bound or bedridden	2.0 (1.4–2.7); <0.001	1.4 (1.1–1.8); 0.002
Urinary incontinence	1.6 (1.2–2.2); 0.004	1.6 (1.3–2.0); <0.001
Bladder catheter	2.3 (1.1–4.8); 0.023	2.8 (1.6–4.8); <0.001
Recurrent urinary infection	1.8 (1.1–2.8); 0.019	1.9 (1.3–2.7); <0.001
Decubitus or skin ulcers	1.8 (1.1–3.0); 0.023	2.6 (1.8–3.7); <0.001
Surgical or other wounds	1.7 (1.0–3.0); 0.068	1.6 (1.0–2.5); 0.029
Percutaneous gastrostomy	1.8 (0.6–5.3); 0.262	2.3 (1.1–5.0); 0.026
Nasogastric tube feeding	1.4 (0.3–5.9); 0.678	2.6 (1.1–6.6); 0.038
Previous known MRSA carriage	2.1 (1.2–3.7); 0.013	4.8 (3.2–7.1); <0.001
Previous known MRSA infection	3.2 (1.7–6.4); 0.001	4.8 (2.8–8.2); <0.001
Previously known ESBLE carriage	8.8 (1.5–52.8); 0.018	1.7 (0.2–14.9); 0.652
Previous hospitalization in the past year	1.5 (1.1–2.0); 0.009	1.1 (0.9–1.4); 0.448
Previous hospitalization for infection in the past year	1.7 (0.9–3.4); 0.112	2.6 (1.6–4.2); <0.001
Antacid use	0.9 (0.7–1.3); 0.570	1.5 (1.2–1.9); <0.001
Current antibiotic use at the time of screening	1.9 (1.1–3.5); 0.031	1.9 (1.2–2.9); 0.008
Previous antibiotic use (<3 months)	1.8 (1.3–2.5); <0.001	1.4 (1.1–1.8); 0.009
Beta-lactam penicilline (JO1C)* use (<3 months)	1.9 (1.2–2.8); 0.004	1.5 (1.1–2.1); 0.019
Amoxicillin-clavulanate (JO1CR) use (<3 months)	2.0 (1.2–3.3); 0.006	1.7 (1.1–2.5); 0.014
Ciprofloxacin (JO1MA02) use (<3 months)	1.4 (0.7–2.9); 0.391	1.9 (1.1–3.1); 0.017
Levofloxacin (JO1MA12) use (<3 months)	3.6 (1.0–13.0); 0.051	1.5 (0.4–5.4); 0.509
Cotrimoxazole (JO1EE01) use (<3 months)	0.8 (0.2–3.5); 0.808	4.0 (2.0–8.0); <0.001
Lincomycin/clindamycin (JO1FF) use (<3 months)	3.6 (1.0–13.0); 0.051	1.0 (0.2–4.5); 0.983
More than 3 antibiotics in the past 3 months	2.1 (1.2–3.7); 0.020	1.8 (1.2–2.9); 0.009
Systemic disease	0.8 (0.3–2.0); 0.621	1.8 (1.0–3.0); 0.033
Chronic obstructive pulmonary disease	1.7 (1.1–2.7); 0.016	1.3 (0.9–1.9); 0.098
Peptic ulcer	0.4 (0.2–0.9); 0.030	1.2 (0.8–1.8); 0.304

MRSA: methicillin-resistant *Staphylococcus aureus*; ESBLE: extended-spectrum beta-lactamase-producing Enterobacteriaceae; OR: odd's ratio;

* classification according to WHO ATC system (http://www.whocc.no/atc_ddd_index/).

## Results

### Included nursing homes and residents

Among 60 randomly selected NHs, 41 accepted to participate. The remaining 19 NHs were recruited from the list of Belgian high-skilled NHs taking into account criteria for region distribution, NH size and proportion of high-skilled NH beds. A total of 2791 residents were screened for at least one of the three targeted MDROs. Screened residents for whom a questionnaire was missing were excluded from analysis. The median age of the study population was 86 years (Interquartile range [IQR]: 81–90 years). The median length of stay in the NHs was 30 months (IQR: 12–61 months). Detailed characteristics of the included residents are presented in Table 2.

**Table 2:** Characteristics of included NH residents (n = 2791) in a point-prevalence survey in 60 NHs in Belgium in 2011.

Characteristics	Subcategory	Result
Age, years; median (IQR) [Range]		86 (81–90) [43–106]
Female/Male gender, n (%)		2128 (77.7)/611 (22.3)
LOS of the resident in the NH, months; median (IQR) [Range]		30 (12–61) [0–353]
Number of patients in a single bed room, n (%)		2127 (76.2)
Level of autonomy according to the modified Katz scale, n (%)	Category O*	383 (13.9)
	Category A	436 (15.8)
	Category B	703 (25.5)
	Category C or CD*	1235 (44.8)
Mobility level, n (%)	Ambulant	1430 (53.3)
	Wheelchair-bound or bedridden	1255 (46.7)
Charlson's Comorbidity Index, n (%)	None or mild	755 (32.2)
	Moderate	1288 (54.9)
	Severe	305 (13.0)
Previous hospitalization in the year before the survey, n (%)		838 (30.0)
	Surgical unit	145 (17.3)
	Intensive care unit	6 (0.7)
	Medical unit	156 (18.6)
	Geriatric ward	326 (38.9)
	Others or unknown	205 (24.5)
Previously known dementia, n (%)		1202 (51.2)
Previously known MRSA colonization, n (%)		109 (3.9)
Previously known ESBLE colonization, n (%)		5 (0.2)
Chronic wounds (decubitus ulcers, surgical wound, trauma), n (%)		305 (10.9)
Chronic catheters (urinary, vascular or gastrostomy), n (%)		127 (4.6)
Naso-gastric tube feeding, n (%)		21 (0.8)
Haemodialysis, n (%)		11 (0.4)
Current MRSA decolonisation procedure at the time of survey, n (%)		3 (0.1)
Current antibiotic use at the time of the survey, n (%)		117 (4.2)
Previous antibiotic use in the past three months, n (%)		599 (21.5)
Antacid use at the time of screening, n (%)		888 (32.8)
Chronic corticoid use or chemotherapy, n (%)		78 (2.8)

* Category O = complete autonomy and Category C or CD = high level of dependency; LOS = length of stay; IQR = interquartile range; MRSA = methicillin-resistant *Staphylococcus aureus*; ESBLE = extended-spectrum β-lactamase-producing *Enterobacteriaceae*.

### Prevalence of colonization

Among the 2791 residents screened for ESBLE carriage, 181 rectal swabs were rejected because not yielding any bacterial growth on McConkey agar control plate. Of the remaining 2610 screened residents, 186 rectal swab samples grew with one or more ESBLE isolates. The weighted prevalence of ESBLE carriage was 6.2% [95% CI: 5.6–6.9], ranging between 0 to 20% in the different NHs. The prevalence was neither different between “low care” and “high care” NHs, nor according to the number of beds or the social status (private or public) of the NHs (data not shown). The mean weighted prevalence of ESBLE carriage was significantly higher in Brussels (11.0% [95%CI: 8.5–14.0]) than in Wallonia (5.1% [95%CI: 4.2–6.2]; *P* = 0.01) and Flanders (6.0% [95%CI: 5.2–6.9]; *P* = 0.037). Among the 2789 residents screened for MRSA, 366 were found to carry MRSA (weighted prevalence: 12.2% [95% CI: 11.3–13.1]). As for ESBLs, the prevalence of MRSA carriage was found to differ widely between NHs ranging from 0 to 36%. The prevalence of MRSA carriage did not differ according to the number of beds in the NH, but was significantly lower among “high care” NH compared to “low care” NH (9.7% [95%CI: 8.1–11.5] and 16.1% [95%CI: 14.2–18.3] respectively; *P*-value: 0.03). The mean weighted prevalence of MRSA was significantly lower in Flanders (7.9% [95%CI: 7.0–8.9]) compared to Brussels (14.7% [95%CI: 11.8–18.1]; *P* = 0.01) or Wallonia (18.3% [95%CI: 16.5–20.2]; *P* = 0.001). No single case of VRE was found during the study. The rate of co-colonization with both ESBLE and MRSA among the 2609 patients was low (25 cases; weighted prevalence 0.8% [95%CI: 0.6–1.1]) and no relationship was found between the prevalence of ESBLE and of MRSA in the NHs.

In order to assess the occurrence of potential recruitment bias, we compared the observed prevalence rates according to the method of recruitment (random selection or active/selected recruitment) and found that the results of prevalence were not statistically different neither for ESBLE (random selection: 5.9% [95%CI: 4.6–7.3]; active recruitment: 6.3% [95%CI: 5.5–7.2]) nor for MRSA (random selection: 14.2% [95%CI: 12.3–16.1]; active recruitment: 11.5% [95%CI: 10.0–13.1]).

### Microbiological data

Among the 2789 residents screened for MRSA, 2789 samples were pooled specimens from anterior nares, throat and perineum while 154 (5.5%) samples were obtained from wounds. Overall, 205 ESBLE isolates were cultured from rectal swabs including *Escherichia coli* (n = 183), *Klebsiella pneumoniae* (n = 10), *Enterobacter aerogenes* (n = 6), *Enterobacter cloacae* (n = 5) and *Citrobacter freundii* (n = 1). Sixty-nine percent of the ESBL-producing strains displayed co-resistance to ciprofloxacin, 54% were resistant to cotrimoxazole and 23% were resistant to gentamicin. None of these 205 *Enterobacteriaceae* isolates displayed reduced susceptibility to meropenem or to ertapenem.

Among *Escherichia coli* strains (n = 183), the most frequently ESBL coding genes were CTX-M of group 1 (n = 106) followed by CTX-M of group 9 (n = 31), TEM-type (n = 32), CTX-M of group 2 (n = 5), and SHV-type (n = 3). Four CTX-M of group 9 and two CTX-M of group 2 *E. coli* strains were found to carry simultaneously a CMY-2 plasmidic AmpC coding gene. In two *E. coli* isolates, no ESBL coding genes could be detected by molecular testing despite a positive double disc synergy test result with clavulanic acid, hence suggesting the possible presence of ESBL not targeted by the Check-MDR CT101 ligase-PCR assay. *Klebsiella pneumoniae* (n = 10) harboured CTX-M of group 1 (n = 5), SHV-ESBL (n = 5), and TEM-ESBL (n = 1). All *Enterobacter cloacae* and *Citrobacter freundii* carried a CTX-M of group 9, while *Enterobacter aerogenes* strains either carried TEM- (n = 4) or SHV- ESBL coding genes (n = 1).

### Risk factors of colonization

Significant risk factors of carriage of ESBLE and MRSA in univariate analysis are shown in Table 1. Using random-effect logistic regression (Table 3), the best predictors of being colonized by ESBLE were previously known ESBLE carriage, a low level of mobility, previous antibiotic exposure in the past three months and male gender. Length of stay in the NH and housing in a non-single bed room were not associated with an increase ESBL carriage risk. The proportion of the total variance contributed by the NH level variance component was low (ICC: 0.05; *P*-value: 0.022). The best predictors of MRSA carriage were (Table 4): previously known MRSA carriage, decubitus ulcer or chronic wounds, a low level of autonomy and antacid use. Compared to ESBLE carriage, the variation in the MRSA risk was more explained by the grouping of residents in single NHs (ICC: 0.11; *P*-value<0.001). Risk factors of co-colonization by both MRSA and ESBLE were bladder catheter (OR: 6.29 [95%CI: 2.61–15.14]; *P*<0.001) and decubitus ulcers (OR: 9.04 [95%CI: 3.15–25.91]; *P*<0.001).

**Table 3:** Multilevel logistic regression analysis of individual risk factors of ESBLE carriage in residents of 60 NHs in Belgium in 2011.

Predictors of ESBLE carriage (n = 2457)	Subcategory	Adjusted OR (95%CI)	*P*-value
Previously known ESBLE carriage	No	1.0 (Reference)	
	Yes	7.8 (1.2–50.6)	<0.031
Mobility impairment (wheelchair bound or bedridden)	No	1.0 (Reference)	
	Yes	1.8 (1.3–2.5)	<0.001
Antibiotic exposure in the past 3 months	No	1.0 (Reference)	
	Yes	1.7 (1.2–2.2)	0.005
Gender	Female	1.0 (Reference)	
	Male	1.5 (1.1–2.1)	0.020

**Table 4:** Multilevel logistic regression analysis of individual risk factors of MRSA carriage in residents of 60 NHs in Belgium in 2011.

Predictors of MRSA carriage (n = 2600)		Adjusted OR (95%CI)	*P*-value
Previous MRSA carriage	No	1.0 (Reference)	
	Yes	3.5 (2.2–5.6)	<0.001
Pressure sores	No	1.0 (Reference)	
	Yes	1.7 (1.1–2.6)	0.013
Antacid use	No	1.0 (Reference)	
	Yes	1.5 (1.2–2.0)	0.001
Katz scale category (level of autonomy)			<0.001
	O	1.0 (Reference)	
	A	1.8 (1.1–2.9)	
	B	1.5 (0.9–2.3)	
	C	2.2 (1.3–3.7)	
	CD	2.3 (1.5–3.6)	

## Discussion

This represents the first large scale study in Europe that included the screening of ESBLE, MRSA and VRE concomitantly in a large sample size of NH residents using standardized microbiological methods. Furthermore, very few studies have used multilevel regression logistic analysis in order to take into account the potential cluster effect of MDRO carriage within NHs as we did. In the present survey, the prevalence of asymptomatic ESBLE and MRSA carriage reached 6.2% and 12.2%, respectively and the rate of simultaneous co-colonization by these two multidrug-resistant bacteria was below 1%. Previous studies performed in other European countries have reported a broad range of prevalence of MDRO. For example, in Northern Ireland and in Italy, high rates of asymptomatic carriage of ESBLE were reported (41 and 64%, respectively) while recent study in Sweden and in France, prevalence rates of 3% or lower have been reported [9], [14], [15], [16]. Variations in the screening sampling sites and in the microbiological methods, differences in the definitions of criteria for the targeted microorganisms, differences in the population case-mix and in local practices as well as true epidemiological variations may probably altogether explain this large variability across countries.

Functional status and previously known MDRO carriage appeared as common risk factor both for ESBLE and MRSA carriage as it was suggested in previous small scale studies [17], [18]. For example, Pop-Vicas *et al.* in a cross-sectional survey conducted within 4 units in a 648-bed long-term care facility in Boston, found that 51% and 28% of screened residents were colonised by multidrug-resistant gram negative bacteria and MRSA, respectively [18]. Advanced dementia and non-ambulatory status were two independent predictors after multivariate adjustment. A reciprocal relationship between infection and functional impairment was reported in a 6-month prospective cohort study within 39 NHs in Switzerland [19]. After adjustment for baseline characteristics, subjects with infection had higher odds of functional decline during two follow-up periods of 3 months in a stepwise fashion while subjects with moderate or severe functional impairment at baseline had a stepwise greater likelihood of infections in survival analysis predicting time to first infection. Functional status is a good surrogate marker of frailty as it results from the interaction between age, disabilities, chronic disease, individual and contextual factors. The evaluation of the functional status is a core component of the comprehensive geriatric assessment while it is usually not considered as a standard of care among conventional medical/surgical units. All these data suggest that the functional status should be assessed in the medical evaluation of patients at risk for colonization or infection by MDRO.

We hypothesize that a low level of autonomy is associated with a higher prevalence of potential risk factors for acquisition of MDRO, a higher risk for cross-transmission and also of antibiotic exposure. The fact that a high level of dependency most of the time constitutes a non-reversible risk factor implies that aged impaired patients require particular attention during medical care and nursing procedures. These findings also underscore the importance of appropriate and timely communication of MDRO carriage status upon inter-facility transfer of NH residents.

We searched for potential effect modifiers between mobility, antacid use or male gender and other exposures but did not found statistically significant interactions or any confounding factors. Male gender after adjustment for previous ESBL carriage, level of mobility and previous antibiotic exposure in the past three months remained a significant risk factor of ESBLE carriage. Compared to women, male residents tended to be younger and less functionally impaired in our cohort while their level of comorbidities (assessed by the Charlson's Comorbidity Index) and the proportion of residents with multiple hospital stay in the past year before the survey were higher. For example, the proportion of male residents with hemiplegia, cerebro-vascular disease and chronic lung disease was higher and tended to be associated with an increased risk of ESBLE carriage in univariate analysis. The small number of included residents in each subgroup may have precluded to reach sufficient statistical power. Furthermore, due to the restricted number of potential predictors that could have been included in the multivariate analysis (no more than 18–20 predictors), we did not adjust for those potential determinants. Other potential risk factors not included in the analysis in this survey (e.g: the role of antibiotic exposure in the previous past year (instead of three months) or previous contact with the hospital in the past years) may also have accounted for the increased prevalence of ESBLE carriage among male residents.

Surprisingly, antacid use appeared a risk factor for MRSA carriage in our NH study. We looked for potential interaction or confounding factors but the only positive association was that the proportion of MRSA carriers among antacid users compared to non-users was higher among patients who had received three or more courses of antibiotic treatments within three months before admission (29 versus 14%; OR: 2.47 [95%CI: 0.90–6.91]) compared to patients with 2 or less antibiotics regimens (16 versus 12%; OR: 1.42 [95%CI: 1.11–1.81]). However, this difference was not statistically significant and confirmation of this hypothesis would require the assessment of a larger cohort of residents. Another plausible explanation for the higher MRSA prevalence in this subgroup of residents could be that antacid users had been more frequently in contact with the healthcare system more than one year before the survey (our defined window when collecting data). While the association of gastric-acid suppression and community-acquired *Clostridium difficile*-associated disease is well known in the literature, the use of antacids has also been linked with community-acquired pneumonia and less frequently with Campylobacter gastroenteritis [20], [21]. Gastric suppression increases the risk of bacterial overgrowth and pharmacological suppression of gastric acid production may also modulate the host immune response and favour immunosuppression [22], [23]. From a practical point, these data support the fact that physicians may have to reconsider the long-term administration of antacid agents, particularly among old frail polymedicated NH residents.

In our study, comorbidities assessed and scored by the Charlson's Comorbidity Index were not found to be significantly associated with the risk of MDRO carriage. This index scale was chosen in order to allow comparisons since it had already been used in a similar survey conducted in 2005, allowing comparison [7]. However, the performance of comorbidity indices among old frail patients who suffer from multiple comorbid conditions has been questioned in the medical literature. The Cumulative Illness Rating Scale-Geriatrics and the Geriatric Index of Comorbidity were reported to better predict, than the Charlson's Comorbidity Index, adverse outcomes after hospital discharge [24]. When considering specific comorbidities, chronic obstructive pulmonary disease was shown as a risk factor for ESBLE carriage probably because patients suffering from this condition are frequently admitted to hospital and exposed to antibiotics. On the other hand, peptic ulcer was found to be protective for ESBLE carriage in univariate analysis but this association was no more significant after adjustment.

Our risk factor analysis suggests that cross-transmission plays a greater role in the epidemiology of MRSA as also suggested by the higher cluster effect and by the fact that 9% of participating NHs reported a MRSA outbreak in the previous year before the survey (data not shown). On the other hand, the cluster effect was less important for ESBLE, mainly ESBL-producing *E. coli* suggesting that cross-transmission could play a less important role compared to that one observed for previous antibiotic exposure, as observed in this study as well as in previous reports [18], [25]. This statement is also supported by several reports that have suggested that patient-to-patient cross-transmission of ESBL- *E. coli* may occur much less frequently than it is observed with other multi-resistant gram-negative nosocomial pathogens (e.g.: *Klebsiella* spp., *Enterobacter* spp.) [26], [27]. However, it should be stressed that the scope of our microbiological investigations was not aimed to directly assess the role of cross-transmission and that we did not include data over the environment, food and healthcare workers. We acknowledge that the low prevalence of ESBLE in this cohort of NH residents, the limits of the screening diagnostic method that was used (see below), the lower sensitivity of ESBLE detection in residents not exposed to antibiotics altogether may have led to underestimation of the potential role of cross-transmission.

Another finding of the study was that the distribution of the ESBLE types closely paralleled that reported in Belgian hospitals, being significantly higher in the Brussels area than in Wallonia and in Flanders [28]. In a continuous nationwide surveillance programme performed within 100 hospitals, the proportion of CTX-M-producing *E. coli* reached 77% in 2008 [29]. Also similar regional variations in prevalence were found, the region with the highest observed ESBLE prevalence among NHs (i.e: in the Brussels area) also corresponding to the one with the highest incidence of ESBLE within hospitals [29]. These results altogether suggest a close relationship between NH and hospital epidemiology most probably through the frequent inter-facility transfer of patients and of NH residents between these two medical sectors.

Our study showed a substantial decrease in the prevalence rate of MRSA carriage between 2005 and 2011. Since 2003, many large scale initiatives have been implemented in Belgium in order to reduce the spread of MDRO. These include for instance, the publication of national guidelines for controlling the transmission of MRSA and ESBLE, national campaigns for the improvement of hand hygiene and national surveillance programmes of antibiotic use within hospitals as well as in the community [30], [31], [32], [33], [34]. We hypothesize that the sequential implementation of all these multifaceted surveillance and interventions within hospitals and in NHs altogether probably did contribute to the decrease in prevalence of MRSA carriage among NH residents. Another factor might have been the significant decrease of antibiotic exposure of NH residents (decrease of 10% in the overall defined daily dose observed in NH residents in 2011as compared to 2005 data) (B. Jans, personal communication).

However, those results have also to be interpreted along the more global trend observed in the EARSS/EARS-Net surveillance programme where a decrease of the proportion of MRSA strains and an increase of ESBLE among bloodstream infections were reported in many European countries including Belgium [35].

We do acknowledge the fact that the study had some limitations. While the sampling site strategies and the laboratory detection methods are well known for MRSA and for VRE detection, there is currently still a lack of knowledge concerning the best screening methods of ESBLE [36]. Stool culture is considered as the gold standard for the detection of ESBLE but it is impractical and difficult to implement on a large scale in epidemiological surveillance programmes. Lautenbach *et al.* have compared the performance of rectal and perirectal swabs to stool culture taken as the gold standard for the detection of fluoroquinolone-resistant *E. coli* [36]. They reported sensitivities and specificities of 90% and 100%, respectively but the 95%CI was large ranging between 70–99% due to the small sample size. False-negative rectal swab results were observed among patients with stool culture with less than 5 colonies per plate. The same authors also found that subjects were frequently colonized by more than one strain of *E. coli* and that the ability of rectal swabs to accurately detect and characterized the diversity of *E. coli* strains from faecal samples was directly related to the number of sampled colonies and the underlying prevalence of the strain [37]. Furthermore, the best screening strategies (one site versus multisite screening approach) remains to be determined and well-powered studies are still required to assess the performance of rectal swabs.

Also, the pattern of ESBLE colonization (i.e.: transient versus chronic carriage) is still poorly known and the cross-sectional design of our survey did not address the dynamics of ESBLE carriage [38]. Data from prior studies have suggested that gut colonization could change over time and that it may be markedly influenced through antibiotic selection pressure [39]. For example, among a cohort of 33 NH residents in Boston who were screened serially every 3–4 weeks by rectal surveillance swabs, the median duration of colonization by multidrug-resistant gram-negative bacteria was 144 days (with range from 41 to 349 days). Clearance of colonization occurred in 39% of episodes. It should be mentioned that factors that are responsible for short-term or long-term carriage are poorly understood. The variable character of ESBLE carriage may have led to underestimation of prevalence and possibly also it may have influenced our conclusions about the potential role of cross-transmission which seems less important in comparison to antibiotic selection pressure.

All, these factors may have influenced the reported prevalence in our study. A second limitation was that nearly one third of participating NHs were actively recruited by phone call through the investigators, taking into account the geographic location, number of beds and proportion of high-skilled beds. However, we did not observe differences of prevalence according to random or active recruitment method and therefore assume this selection bias as minimal. A third limit of the study was the lack of screening for carbapenem-resistant *Enterobacteriaceae* while their spread was recently reported in Belgian hospitals [40]. We decided not to include carbapenem-resistant *Enterobacteriaceae* because of the limits of current detection methods, especially the low sensitivity of several commercial agar plates to detect low level expressed OXA-48 producing carbapenemases, as reported recently in a thorough review [41].

In practice, these data support all the past and current efforts to limit the usage of broad-spectrum antibiotics and to follow national guidelines for empirical therapy [42]. Clinicians must include in the decision process, the local epidemiology, a previous alert for infection or colonisation by MDRO, the number of potential risk factors and the severity of illness before prescribing antibiotic treatment.

## Conclusion

The prevalence of asymptomatic carriage of ESBLE was found to be low in a large cohort of nursing home residents in Belgium, though with a wide prevalence range being observed between facilities, and that the proportion of MRSA did markedly decrease by 6.8% since the former study in 2005. The latter trend is in line with the nationwide evolution of MRSA infections in acute care hospitals. No cases of VRE were found. Overall, these results are highly encouraging and support further maintaining all initiatives that have been implemented at national level to reduce the spread of MDRO within and across acute and chronic health care facilities.
